# The temperature dependence of the Hildebrand solubility parameters of selected hydrocarbon polymers and hydrocarbon solvents: a molecular dynamics investigation

**DOI:** 10.1007/s00894-024-05929-w

**Published:** 2024-06-05

**Authors:** Gabriel P. Costa, Phillip Choi, Stanislav R. Stoyanov, Qi Liu

**Affiliations:** 1 Department of Chemical and Materials Engineering, University of Alberta, Edmonton, AB T6G 2G6 Canada; 2 Faculty of Engineering and Applied Science, University of Regina, Regina, SK S4S 2A0 Canada; 3 Natural Resources Canada, CanmetENERGY Devon, 1 Oil Patch Drive, Devon, AB T9G 1A8 Canada

**Keywords:** Hildebrand solubility parameter, Non-polar polymer, Temperature dependence of polymer solubility, Glass transition temperature, Molecular dynamics simulations

## Abstract

**Context:**

To determine the miscibility of liquids at high temperatures using the concept of Hildebrand solubility parameter $\delta$, the current practice is to examine the difference in $\delta$ between two liquids at room temperature, assuming that $\delta$ is not sensitive to temperature*.* However, such an assumption may not be valid for certain polymer blends and solutions. Therefore, a knowledge of the *δ* values of the liquids of interest at high temperatures is desirable. The determination of *δ* at high temperatures, especially for high-molecular-weight polymers, is impossible, as polymers have vapor pressures of zero. To this end, molecular dynamics (MD) simulations provide a practical means for determining *δ* over a wide range of temperatures. In this work, we study the temperature dependence of $\delta$ of five hydrocarbon polymers: polyethylene (PE), isotactic and atactic polypropylene (*i*-PP and *a*-PP), polyisobutylene (PIB), and polyisoprene (PI) in five hydrocarbon solvents: *n*-pentane, *n*-hexane, *n*-dodecane, isobutene, and cyclohexane. The polymers are modeled as monodisperse chains with 100 repeat units. The average *δ* values of PE, *i*-PP, *a*-PP, PIB, and PI at 300 K are determined as 18.6, 14.9, 14.6, 14.3, and 16.4 MPa^1/2^, respectively, in a good agreement with experimental data. The *δ* values of these polymers at various temperatures are also determined. The temperature dependence of *δ* is fitted to two linear equations, one above and the other below the polymer’s glass transition temperature *T*_*g*_. The *δ* values are more sensitive to temperature at *T* ≥ *T*_g_. The *T*_g_ values of the polymers, determined based upon their specific volumes and *δ* values agree with the experiment qualitatively. The determination of the temperature dependence of *δ* has a great potential for industrial applications, such as determining miscibility, developing polymeric organogelators as flocculants and oil spill treating agents, and identifying potential solvents and ideal processing temperatures.

**Methods:**

The MD simulations are performed using the GROMACS 2022.3 package with optimized potential for liquid simulations-all atom (OPLS-AA) force field parameters. All polymers are built as extended chains using CHARMM-GUI Polymer Builder.

**Graphical Abstract:**

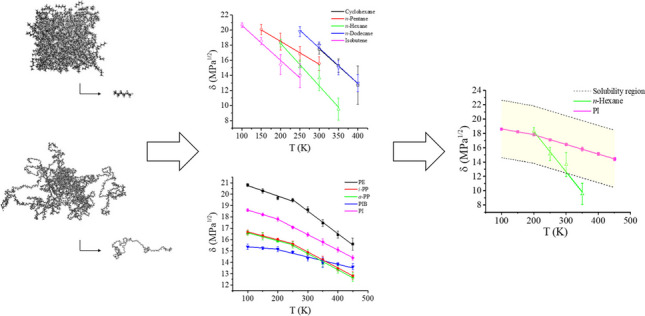

## Introduction

The Hildebrand solubility parameter, *δ*, is a property that characterizes the strength of intermolecular forces between molecules in a pure liquid. Liquids with similar *δ* values (i.e., $\Delta \delta ={\delta }_{1}-{\delta }_{2}$ ~ 0) are expected to be miscible into each other. Therefore, *δ* is commonly used to predict the solubility of a polymer in a specific solvent and the miscibility of two polymers. This is essentially the rule of “like dissolves like” [1–3]. The solubility parameter is also used to determine the activity coefficients of components in binary solutions. Other thermodynamic properties, such as glass transition temperature, *T*_g_, and surface tension, γ, are considered to be related to *δ* [3, 4]. Certain established industrial sectors use *δ* frequently. For example, the coatings industry uses *δ* for the selection of appropriate solvents for polymers used in coating formulations. Solubility parameters can be used to predict the likelihood of organogel formation and to help develop polymeric gelators for oil spill treatment and flocculation of clay minerals. Lastly, applications involving polymer blends and polymer thin films also use *δ* to predict compatibility, permeation, and swelling [5–7].

The Hildebrand solubility parameter, *δ*, is defined as the square root of the cohesive energy density (CED) at room temperature, as shown in Eq. 1. The CED equals the ratio of the internal energy change of vaporization ($\Delta {U}_{vap})$ to the molar volume (${V}_{l}$) of a liquid [8, 9], as shown in Eq. 2. The CED essentially is the energy required to vaporize one mole of liquid molecules to vacuum, or the ideal gas state, in which molecules experience no intermolecular forces. The evaporation to the ideal gas state leads to Eq. 3.

1 $$\delta =\sqrt{CED}$$

2 $$CED=\frac{\Delta {U}_{vap}}{{V}_{l}}=\frac{\Delta {H}_{vap}-{\text{RT}}}{{V}_{l}}$$

3 $$\Delta {U}_{vap}=\Delta {H}_{vap}-RT$$

Considering that $\Delta {U}_{vap}$ and ${V}_{l}$ naturally decrease and increase, respectively, as the temperature is increased, it is expected that *δ* would decrease as the temperature is increased.

Owing to the difficulty of measuring the *δ* values of polymers due to their zero vapor pressures*,* the internal pressure (i.e., ${\left(\frac{\partial U}{\partial V}\right)}_{T}$) is sometime used to approximate CED times a constant *n*.

4 $${\left(\frac{\partial U}{\partial V}\right)}_{T}={T\left(\frac{\partial P}{\partial T}\right)}_{V}-P\approx n\frac{CED}{{V}_{l}}$$

It is worth noting that the internal pressure signifies the volume dependence of the internal energy change of a liquid at constant temperature. Unlike the CED concept, no evaporation is involved in the internal pressure definition. Nonetheless, it has been found experimentally that *n* in Eq. (4) varies from 0.9 to 2.0 for various polymers. The above range of *n* is valid for nonpolar compounds, or even for slightly polar compounds, if strong polar interactions, such as hydrogen bonding, are not present [10–12].

Experimental data on the *δ* values of polymers at temperatures other than room temperature is scanty [13, 14]. Previous research has attempted to address the temperature dependence of *δ*, either by thermodynamic derivations or by employing the equations of state [15–19]. In the case of polymers, Chen et al. [20] summarized the theoretical developments of *δ* as a function of the temperature in Eq. 5 and Eq. 6.

5 $${\delta (T)}^{2}=\frac{\left[{H}_{c}\left({T}_{g}\right){e}^{-4{\alpha }_{l}\Delta T}+R{T}^{2}(\frac{\partial {\text{ln}}{\nu }_{f}}{\partial T})\right]}{{\nu }_{l}\left(T\right)M}, T>{T}_{g}$$

6 $${\delta(T)}^2=\frac{\left[H_c\left(T_g\right)e^{-4\alpha_g\Delta T}-M\int_T^{T_g}\Delta C_p(T)dT\right]}{\nu_s\left(T\right)M},T<T_g$$

Equation 5 is applicable to temperatures above *T*_g_ while Eq. 6 is applicable to temperatures below it. The *H*_c_(*T*_g_) is the molar composite enthalpy at *T*_*g*_, *α* is the thermal expansion coefficient, *R* is the gas constant, *M* is the molar mass, and *ν* is the specific volume. The subscripts *s* and *l* denote the glassy and rubbery states, respectively, and *v*_*f*_ is the fractional free volume.

Although the above equations provide an approach to evaluate *δ* at various temperatures, the number of physical properties required makes it impractical. Barton [18] has been the first to report a linear temperature dependence of *δ* based upon the data of 57 small organic molecules (Eq. 7), where the angular coefficient *m* and the linear coefficient *δ*_ref_, taken as a reference *δ* specific to each compound, are empirically fitted and tabulated.

7 $$\delta ={\delta }_{ref}+mT$$

For polymers, a linear temperature dependence has also been observed in the glassy phase (*T* < *T*_g_) as well as in the rubbery state (*T* > *T*_g_), as described by Eq. 8, where *δ*_g_ is the Hildebrand solubility parameter at *T*_g_. The subscripts *s* and *l* denote the glassy and rubbery states, respectively. The angular coefficients ${m}_{s}$ and ${m}_{l}$ are used at *T* < *T*_g_ and *T* > *T*_g_, respectively. The intersection of the two linear temperature dependences occurs at *T*_g_. The authors also reported that both angular coefficients ${m}_{s}$ and ${m}_{l}$ have negative values, with ${m}_{l}$ being more negative than ${m}_{s}$ [20, 21].

8 $$\delta ={\delta }_{g}+{m}_{s,l}(T-{T}_{g})$$

Equation 8 is simple and easy to use. Once *δ*_g_, ${m}_{s}$, and ${m}_{l}$ for a given polymer are known, *δ* can be readily calculated at any *T*. This is particularly useful for determining the miscibility of two polymers, or the solubility of a polymer–solvent pair. It also provides information on the selection of processing temperature [20, 21].

An extremely useful approach to circumvent the limited experimental data at high temperatures is to determine *δ* by using molecular dynamics (MD) simulations, as their computational cost is relatively low [22, 23]. The calculation of *δ* only involves computing the energy of vaporization that is equal to the difference between the potential energy of a bulk amorphous polymer and its energy in vacuum, as shown in Eq. 9 [24, 25]. Another noteworthy advantage of MD is that unlike experiment, no thermal degradation would occur at high temperatures [26].

9 $$\delta =\sqrt{\frac{\Delta {U}_{vap}}{{V}_{l}}}=\sqrt{\frac{{E}_{vac}-{ E}_{bulk}}{{V}_{l}}}$$

In this work, in order to tackle the limited amount of data available and provide a deeper understanding of the dependence of the Hildebrand solubility parameters on temperatures, MD is employed to simulate a selection of hydrocarbon polymers and small molecules at eight different temperatures, ranging from 100 to 450 K. The solubility parameters are calculated as described by Eq. 9 and the findings shed light on the relation among *δ*, *T*, and $\Delta \delta$ at *T* different from room temperature. The presented approach for determining *δ* can also be applied to determine miscibility as well as to identify potential solvents and ideal processing temperatures.

## Molecular simulation methods

### Bulk amorphous and vacuum simulations

**Fig. 1:**
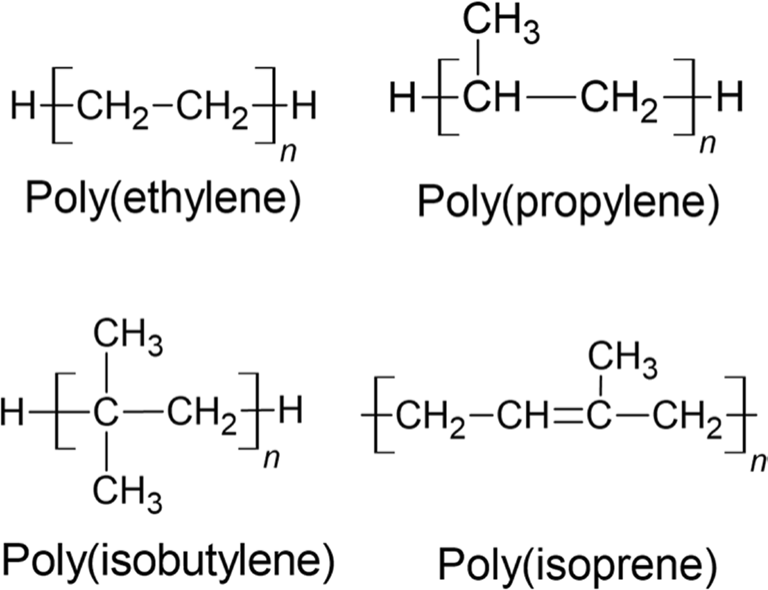
Repeat units of PE, PP, PIB, and PI

Five hydrocarbon polymers were simulated both in the bulk amorphous state and in vacuum. Monodisperse models of polyethylene (PE), isotactic and atactic polypropylene (*i*-PP and *a*-PP), polyisobutylene (PIB), and polyisoprene (PI) were initially simulated in seven different degrees of polymerization (DP), ranging from 10 to 500 repeat units at 300 K. The polymers with 100 repeat units were selected to undergo the temperature dependence evaluation. Eight different temperatures were evaluated, ranging from 100 to 450 K at a 50 K interval, and the simulation details are described below. Cyclohexane, *n*-pentane,* n*-hexane, *n*-dodecane, and isobutane were also simulated both in bulk and vacuum as solvents and small molecules. The model structures of the polymers were originally generated as extended chains using the CHARMM-GUI Polymer Builder [27, 28], as shown in Fig. 1.

In order to maintain an approximate constant cubic cell size, the number of total repeat units in every simulation cell was kept constant. A total of 3000 backbone carbon atoms were present in every polymer bulk simulation. For the small molecules, a fixed number of 500 molecules were present in each simulation cell.

There has been no consensus in the literature on how to perform the vacuum simulations for *δ* calculations. In fact, many works did not describe their vacuum simulations approach [29]. Thus, we found it relevant to briefly outline here what the literature brings as possible options. The most traditional approach would be to simulate a single isolated molecule in a very large simulation cell, taking the extended chain conformation as the input conformation [25, 30]. Zhao and Choi [31] pointed out the different results obtained between this approach and the alternative of taking the input conformation as one of the molecules from the final state of a previous amorphous state production run.

The main problem faced by both approaches is that the isolated molecule was allowed to move freely, which granted it the ability to interact with itself. This might not be relevant to small molecules, but it becomes particularly problematic for macromolecules. Macromolecules can coil and fold, which produces structures that do not reflect the molecule structure in the amorphous state, as required by the definition of *δ*, but it rather denotes how “well dissolved” the polymer is in vacuum (or in other words, how good of a solvent vacuum is for the polymer) [32]. Li et al. [33] suggested that deactivating all intermolecular interactions would result in more realistic calculations. This, although it might differ from one software to another, can be translated into the practical approach of freezing the molecule’s coordinates as they were in the bulk state, which is the equivalent to a 0 K simulation. As the authors demonstrated [29], it prevents coil formation, while retaining the amorphous-state conformation.

Here, a single polymer chain taken from the last state of the bulk simulation was simulated in vacuum with its spatial coordinates frozen. For DPs of 100 and larger, each chain was taken out of the bulk and simulated independently in vacuum. For DPs smaller than 100, the vacuum simulations of polymers were limited to 15 chains, chosen randomly from the bulk, and each polymer was simulated independently in vacuum. These represent 50%, 30%, 20%, and 10% of the total number of chains of the simulations with DP of 50, 30, 20, and 10, respectively. For the small molecules, 50 molecules (10% of the total) were selected randomly, and each one was independently simulated in the vacuum state. The average potential energy, taken as the *E*_vac_, was found to provide enough statistical data.

### Force field and simulation details

The molecular simulations were performed using the GROMACS 2022.3 package [34] with the optimized potential for liquid simulations-all atom (OPLS-AA) force field parameters [35, 36]. Initially, the equivalent number of extended chain molecules were randomly inserted in the simulation cell in order to obtain 6000 backbone carbons according to the DP being studied.

The influence of the chain length on the solubility parameter was evaluated, and a single chain length was chosen for the temperature dependence study. For each DP, the polymer systems were first subjected to energy minimization using the steepest-descent algorithm, for either 50,000 steps or until the maximum force was smaller than 10 kJ/mol^.^nm. The systems were next equilibrated at high temperature using the leap-frog algorithm in a canonical (NVT) ensemble for 100 ps with a time step of 2.0 fs, followed by another equilibration in an isothermal-isobaric (NPT) ensemble for 500 ps with a time step of 2.0 fs, followed by a third equilibration in an NPT ensemble for 100 ns. A Bussi-Donadio-Parrinello (stochastic velocity rescaling) thermostat was used in all equilibration runs to ensure the temperature was kept constant at 500 K. The systems were then annealed down to 300 K at a cooling rate of 10 K/ns. A production run was performed for each DP using the velocity-Verlet algorithm in an NPT ensemble with periodic boundary conditions and a time step of 1.0 fs for 5 ns at 300 K and 1 atm. A Parrinello-Rahman barostat was used to keep the mean pressure constant, while a Nosé-Hoover thermostat was applied to keep the mean temperature constant.

The chain length of 100 repeat units was chosen for the temperature dependence study. The energy-minimized simulation cell was taken from the chain length effect simulations and used as the starting point of the simulations at different temperatures. Three equilibration steps at 500 K were performed using the leap-frog algorithm. The equilibration was performed in three steps, the first in an NVT ensemble for 100 ps with a time step of 2.0 fs, the second, in an NPT ensemble for 500 ps with a time step of 2.0 fs, and the third, also in an NPT ensemble with a time step of 2.0 fs, but for 100 ns. The temperature of 500 K was selected to ensure the chains could freely relax and find the lowest energy state, as it is above every polymer’s melting point. In all simulations, the Bussi-Donadio-Parrinello thermostat was employed. After the equilibration, the systems were annealed down to the desired simulation temperature at a cooling rate of 10 K/ns. The production runs were performed using the velocity-Verlet algorithm in an NPT ensemble with periodic boundary conditions and a time step of 1.0 fs for 5 ns. A Parrinello-Rahman barostat was applied to keep the mean pressure at 1 atm. A Nosé-Hoover thermostat was used to keep the mean temperature at the desired values.

Due to their considerably simpler structure, the small molecule simulations were slightly simpler. In the simulation cell, 500 molecules were randomly inserted. The molecules underwent an energy minimization step, as described above for the polymers, followed by two equilibration steps using the leap-frog algorithm already at the desired temperature. The systems were equilibrated in an NVT ensemble for 100 ps with a 2.0 fs time step, followed by an NPT ensemble for 1 ns with a 1.0 fs time step. The Bussi-Donadio-Parrinello thermostat was used in both equilibration runs. The production run was performed using the velocity-Verlet algorithm in an NPT ensemble with periodic boundary conditions and a time step of 1.0 fs for 1 ns at 1 atm and the desired temperature. The Parrinello-Rahman barostat and the Nosé-Hoover thermostat were employed to keep the mean pressure at 1 atm and the mean temperature at the desired values, respectively.

The electrostatic interactions were calculated using the particle mesh Ewald summation in all simulations [37]. Both Leonard-Jones and electrostatic interactions were truncated at a cutoff radius of 1.0 nm, and only bonds between hydrogen and heavy atoms were constrained. The linear constraint solver (LINCS) algorithm was used to solve all constraints [38].

## Results and discussion

### The chain length dependence

The Hildebrand solubility parameter has been reported to exhibit a chain length dependence, especially at low chain lengths. This dependence has been attributed to the chain ends effect. In this effect, an increase in terminal groups, either by branching or by backbone ends, is responsible for increasing the free volume of the polymer, which depresses some polymer properties, such as *T*_*g*_ and *δ* [39]. To determine which chain length should be used in the desired simulation, it is common to first investigate the chain length dependence. Many authors observed that the *δ* values approached a plateau when the number of repeat units was between 20 and 50. The DP of the plateau was considered to properly represent the polymeric system and a DP value in the plateau range was selected for further simulations [33, 40–42].

**Fig. 2:**
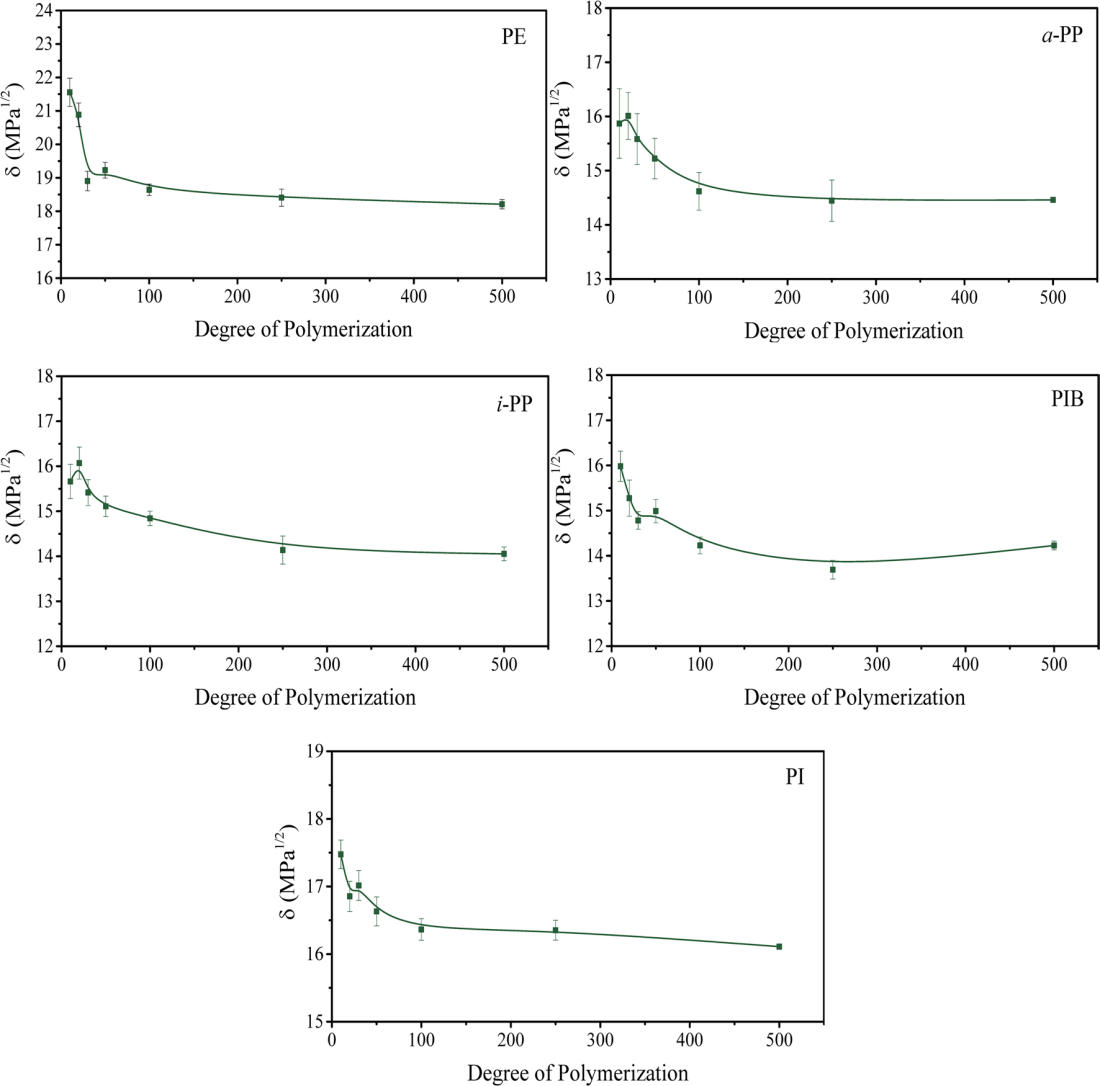
Chain length dependence of the Hildebrand solubility parameters of PE, *i*-PP, *a*-PP, PIB, and PI at 300 K

In our work, for the study of the temperature dependence of *δ*, we first simulate seven different degrees of polymerization for 5 ns at 300 K in order to investigate the chain length dependence and select an adequate DP that represents adequately the polymeric system. The results are presented in Fig. 2. Our results show that the *δ* values decrease rapidly as the degree of polymerization increases, up to the point where it is stabilized between 50 and 100 repeat units. Thus, we select 100 repeat units as representative of our polymers for subsequent calculations.

In Table 1, a comparison of the experimentally determined *δ* values of linear and branched alkanes with those of the polymers PE, PP, and PIB demonstrates that *δ* generally increases as the size of the hydrocarbon molecule is increased. Nonetheless, the behavior observed for our polymers does not reflect this trend. As shown in Fig. 2, the polymers with the lowest DP present the highest *δ* and the latter steadily decrease until reaching a plateau at about 100 repeat units. This is not entirely new behavior and has been observed previously by other authors [42, 43]. One possible explanation is based on the difficulty in equilibrating larger molecules, thus underestimating their solubility parameters [44, 45]. Another possible explanation is that the intramolecular interaction experienced by the polymer in vacuum is greater for longer chains, as these are able to fold and the molecule can interact with itself. Thus, the specific potential energy of a long chain in vacuum and as a result, the calculated solubility parameter would be decreased [33].

**Table 1:** Experimental Hildebrand solubility parameter of alkanes at 298 K from [46–48]

Compound	*δ*_lit_ (MPa^1/2^)	Compound	*δ*_lit_ (MPa^1/2^)
Isobutane	12.8	*n*-Dodecane	16.0
Neopentane	12.9	Cyclohexane	16.7
*n*-Pentane	14.5	Polyethylene	16.9 ± 0.1
*n*-Hexane	14.9	Polypropylene	16.5
*n*-Decane	15.8	Polyisobutylene	16.5

### Hildebrand solubility parameters at room temperature

In order to evaluate the validity of the MD calculations, the results of the simulated polymers at 300 K are evaluated. The solubility parameters were calculated by averaging five vacuum simulations. The *δ* values determined for PE, *i*-PP, *a*-PP, PIB, and PI are 18.6, 14.9, 14.6, 14.3, and 16.4 MPa^1/2^, respectively (Table 2). The common ranges for *δ* of the evaluated polymers, *δ*_lit_, are presented in Table 2. As it can be seen, the calculated *δ* values are in accordance with previously reported ranges in the literature [49–51]. Based on this agreement, our approach and force field are considered suitable for calculation at other temperatures [20, 52].

**Table 2:** Average calculated Hildebrand solubility parameter and the common ranges from Barton [49]

Polymer	Average *δ* (MPa^1/2^)	*δ*_lit_ (MPa^1/2^)
PE	18.6 ± 0.2	14.8 – 19.9
*i*-PP	14.9 ± 0.2	15.5 – 17.5
*a*-PP	14.6 ± 0.2	15.5 – 17.5
PIB	14.3 ± 0.2	14.4 – 17.7
PI	16.4 ± 0.2	16.3 – 17.8

### Hildebrand solubility parameters at elevated temperature

**Fig. 3:**
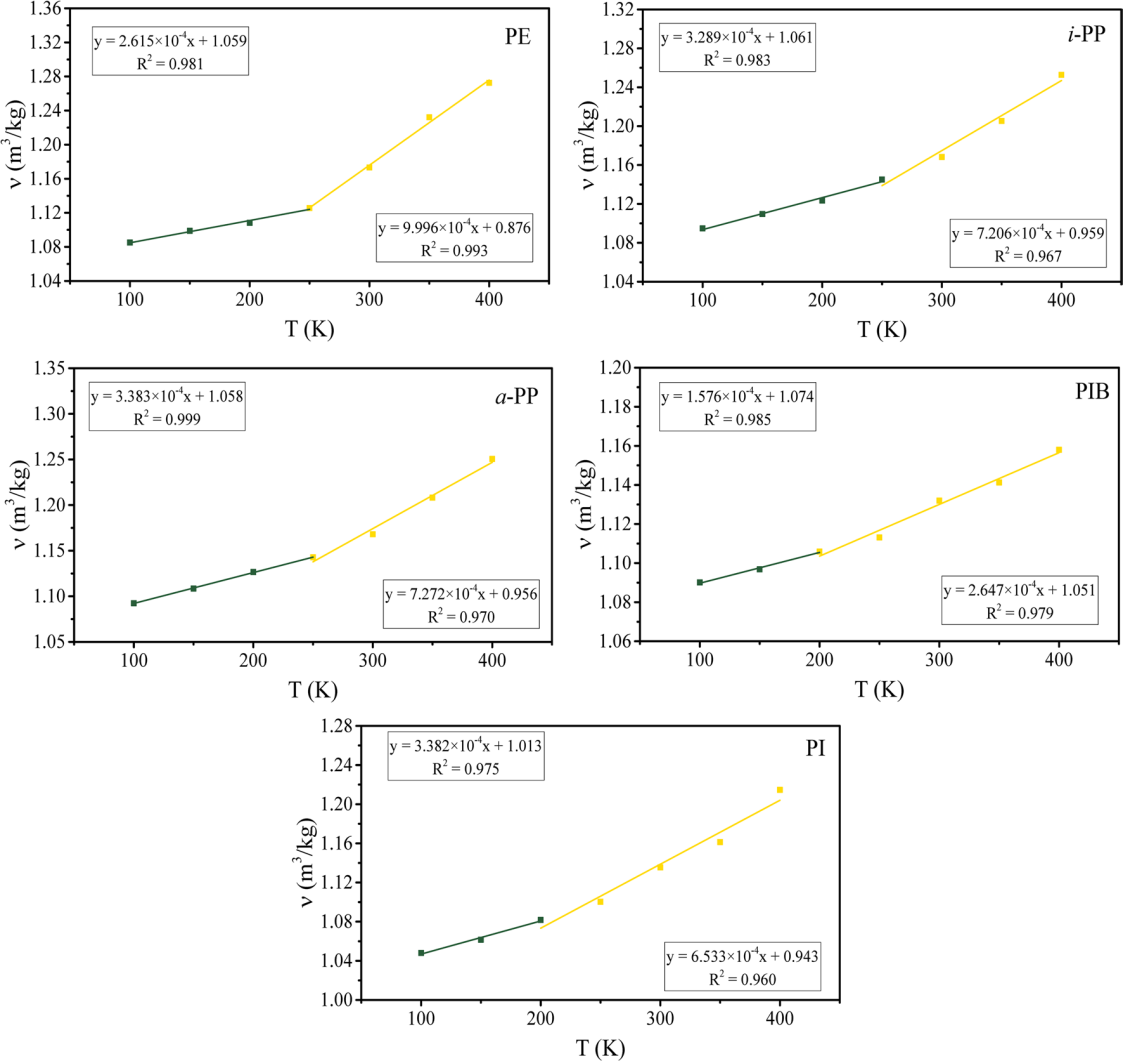
Temperature dependence of specific volume of five hydrocarbon polymers

We also calculate the specific volume of the polymers over a wide range of temperatures that encompass the glass transition temperatures of the polymers [53]. As shown in Fig. 3, there exists a discontinuity in all specific volume versus temperature curves, dividing the plots into two regions in which data points can be fitted by two lines with different slopes. The *T*_g_ is estimated using the intersection of the two best-fit lines, as described in Eq. 8 [33]. This approach has also been reported previously for validation of the calculations [20].

The *T*_g_ values are determined as 248, 260, 262, 214, and 222 K for PE, *i*-PP, *a*-PP, PIB, and PI, respectively. The PE is traditionally reported as having three amorphous phase transitions: the first at 145 K, the second at 195 K, and the third at 240 K. All three temperatures have already been reported as the *T*_g_ of PE [54]. Elastic modulus analysis has shown that the glass transition region of PE is in the range from 170 to 240 K [53]. The *T*_g_ value of 248 K, found in our work, is comparable to those of other computational studies [55, 56]. A range of temperatures have been reported for the *T*_g_ of PP. In particular, the *T*_g_ of PP varies from 255 to 259 K for *i*-PP and from 249 to 267 K for *a*-PP [57, 58]. Once again, the *T*_g_ values reported here agree with the literature values for both *i*-PP and *a*-PP. A common average *T*_g_ value of PIB is about 200 K [59], with a range from 197 to 214 K, depending on the heating rate of the differential scanning calorimetry experiments [60]. It has been observed that MD simulations tend to overestimate it by about 22 K [55]. The value obtained here for PIB is in this reported range. The PI has also a known *T*_*g*_ range of 195 to 211 K [61]. The *T*_*g*_ value obtained here for PI is slightly above the range (222 K); however, it is considered to be in agreement with the literature, as the overestimation is smaller than half of the temperature interval used in this study.

### Temperature dependence

**Fig. 4:**
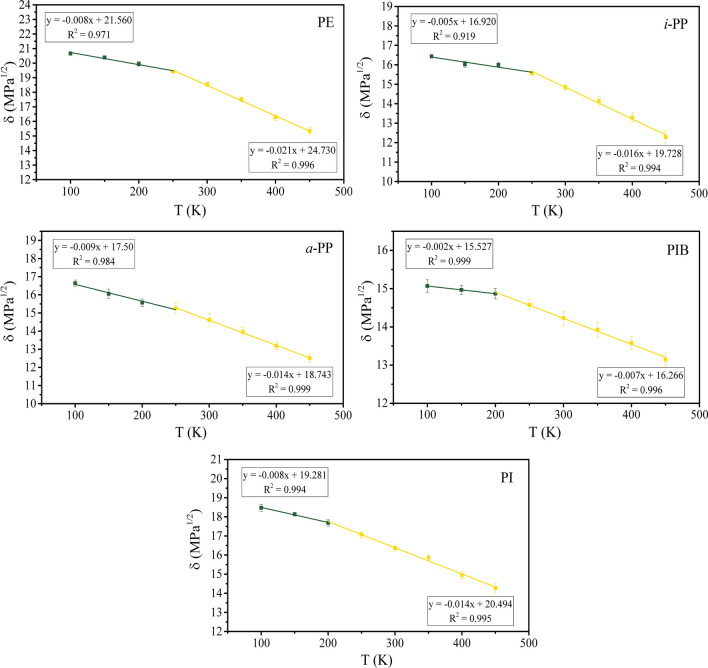
Temperature dependence of Hildebrand solubility parameters of PE, *i*-PP, *a*-PP, PIB, and PI

Figure 4 shows the temperature dependence of *δ* of the five hydrocarbon polymers investigated in this work. Similar to the specific volume (Fig. 3), a discontinuity exists in all plots, leading to two regions, in which two best-fit lines can be obtained below and above the discontinuity. Both best-fit lines have a negative slope with the one above the discontinuity exhibiting a steeper slope.

Table 3 summarizes the values of the slopes before and after the discontinuity (i.e., angular coefficients ${m}_{s}$ and ${m}_{l}$ in Eq. 8). The interception of the two best-fitted lines yields the *T*_*g*_ of the polymer that is also shown in Table 3. These values are comparable to those obtained from the specific volume analysis [20, 53].

**Table 3:** Temperature dependence parameters of Eq. 8 determined from the *δ* versus *T* over the temperature range of 100–450 K

Polymer	${m}_{s}$ (MPa^1/2^ K^−1^)	${m}_{l}$ (MPa^1/2^ K^−1^)	*T*_g_ (K)	*δ*_g_ (MPa^1/2^)
PE	− 0.008	− 0.021	243	19.6
*i*-PP	− 0.005	− 0.016	255	15.6
*a*-PP	− 0.009	− 0.014	248	15.3
PIB	− 0.002	− 0.007	199	15.7
PI	− 0.008	− 0.014	202	17.7

Given that there are no significant changes in the packing of the macromolecules below *T*_g_, it is expected that *δ* should be weakly dependent of temperature. Indeed, for all polymers evaluated, *δ* is approximately constant when *T* < *T*_g_ as supported by extremely low ${m}_{s}$ values obtained in the order of 10^−3^ MPa^1/2^ K^−1^. In particular, PIB was found to have very low ${m}_{s}$ and ${m}_{l}$, indicating a weak dependence throughout the temperature range evaluated. On the other hand, the ${m}_{l}$ values of all other polymers are comparable and on the order of 10^−2^ MPa^1/2^ K^−1^. The values of ${m}_{s}$ and ${m}_{l}$ for PE agree with previous reports [21], while the ${m}_{s}$ and ${m}_{l}$ values for *i*-PP, *a*-PP, PIB, and PI are reported for the first time.

As mentioned, a common assumption of using *δ* in miscibility prediction is that $\Delta \delta =\left({\delta }_{1}-{\delta }_{2}\right)$ is independent of temperature [62]. The compounds are considered miscible when $\Delta \delta =\left({\delta }_{1}-{\delta }_{2}\right)<4 {MPa}^{1/2}$ [1]. Comparable ${m}_{s}$ and ${m}_{l}$ values between two polymers indicate that $\Delta \delta$ can be considered independent of temperature, as the changes associated with it would be minimal.

**Fig. 5:**
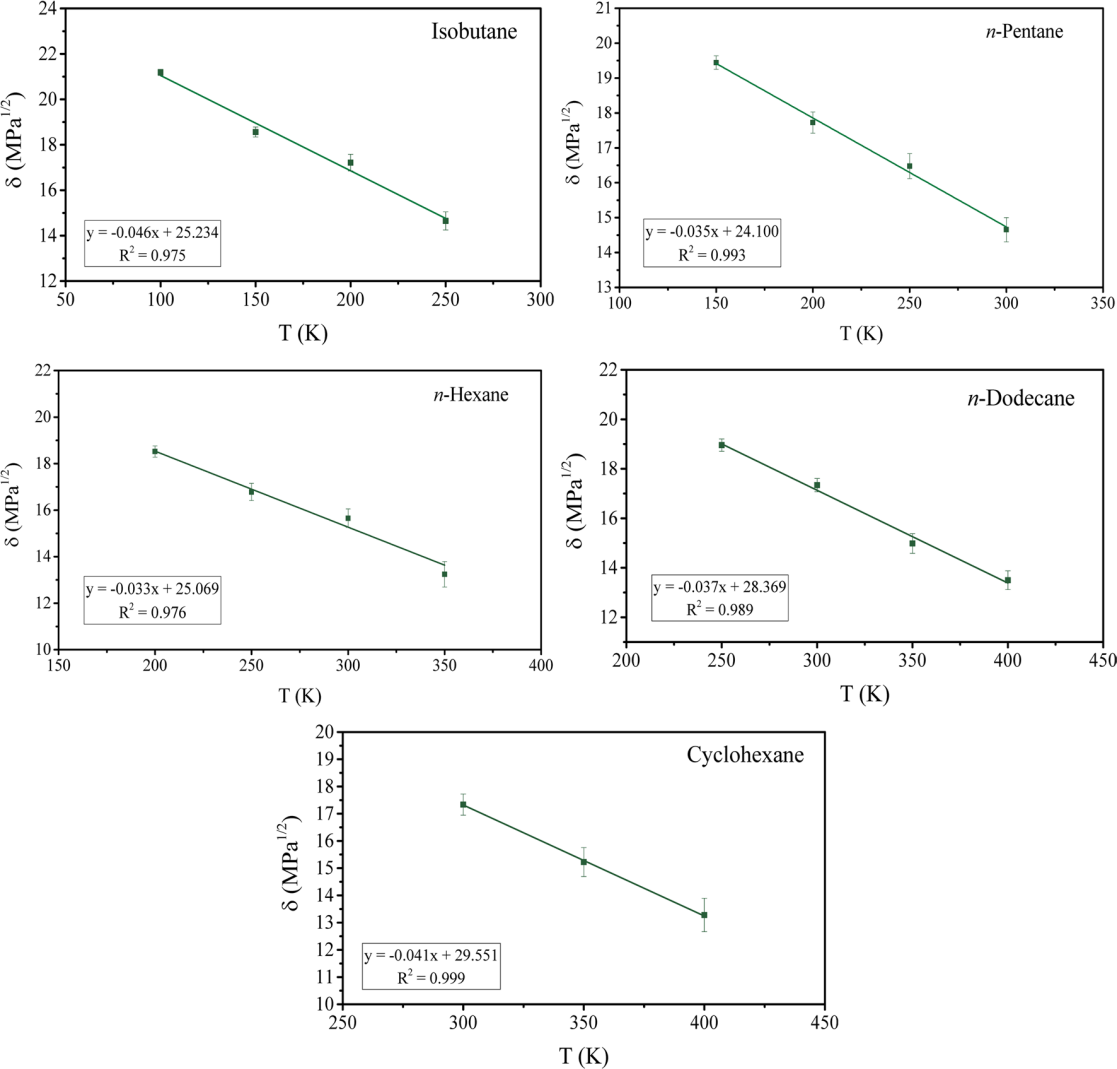
Temperature dependence of Hildebrand solubility parameter of selected small molecules

The same evaluation is performed for *n*-pentane, *n*-hexane, *n*-dodecane, isobutane, and cyclohexane with the goal of comparing the trends observed with solvents/small molecules. A similar behavior is observed, with a discontinuity in the linear trend, dividing the plot into two linear fits. In the case of small molecules, the intersection point of the lines is the melting point. Nonetheless, as in this work, we are interested in polymer solutions, we limit the temperature range to the liquid phase of these compounds only. The solubility parameter analyses are presented in Fig. 5.

All small molecules also have similar ${m}_{l}$ values, as listed in Table 4. Furthermore, the order of magnitude was found to be 10^−2^ MPa^1/2^ K^−1^, similar to the one obtained for the polymers above their *T*_g_. Despite being of the same order of magnitude, the ${m}_{l}$ values of small molecules are 3–4 times greater than the ${m}_{l}$ values of polymers. This suggests that, even though there is a continued growth of $\Delta \delta$ with temperature, the $\Delta \delta$ for polymer solutions will exhibit a weak temperature dependence.

**Table 4:** Temperature dependence parameters for selected small alkanes determined from the *δ* versus *T* over the temperature range of the liquid phase

Polymer	${m}_{l}$ (MPa^1/2^ K^−1^)	*δ*_ref_ (MPa^1/2^)
Isobutane	− 0.046	25.2
*n*-Pentane	− 0.035	24.1
*n*-Hexane	− 0.033	25.1
*n*-Dodecane	− 0.037	28.4
Cyclohexane	− 0.041	29.6

The results presented here, even considering the limited number of hydrocarbon polymers, strongly suggests that the assumption of $\Delta \delta$ being independent of temperature holds true for such polymers and their blends. Moreover, there is a weak temperature dependence for polymer-small molecules pairs. It is worth noting that in order to fully acknowledge the behavior discussed here, more data on a wider range of polymers and small molecules is needed.

## Conclusions

Molecular dynamics simulations were employed to study the temperature dependence of the Hildebrand solubility parameter of five hydrocarbon polymers. The chain length dependence of *δ* was first evaluated and found to be slightly different from what the literature describes as the expected behavior; however, it was also not entirely new, as other works had similar reports, especially for DP ≥ 100. The temperature dependence of *δ* was studied using a chain length of 100 repeat units.

The results show that Eq. 8 described the data well. The glass transition temperatures of the polymers were obtained from the plots generated using Eq. 8 and exhibited a qualitative agreement with the experimental values. The findings suggested that *δ* was insensitive to temperature, when temperature was below *T*_*g*_. Temperatures above *T*_g_ presented a decreasing tendency, similar to the one observed for small molecules with two to three times smaller slope. The slope was found to be five to six times larger below *T*_g_.

The findings corroborated the assumption that $\left({\delta }_{1}-{\delta }_{2}\right)$ could be considered temperature independent, especially for polymer blends. However, this assumption may not be fully suitable for polymer solutions, where a weak temperature dependence was found. The results provided useful insights into the relationship between *δ* and *T*, especially on the effect of temperature in miscibility prediction via $\left({\delta }_{1}-{\delta }_{2}\right)$. These findings highlight the important potential of the temperature dependence of $\delta$ for industrial applications, such as identifying the most adequate blending and processing temperatures. The improved understanding of solubility parameters can help broaden the range of organogelator solvents and provide tools for optimizing the thermal conditions for polymer dissolution and miscibility.

## Data Availability

Datasets can be provided upon reasonable request via email. Please contact G.P. Costa on gpereir1@ualberta.ca.
